# INVESTIGATING THE INVERSE RELATIONSHIP BETWEEN SMOKING AND PARKINSON’S: EVIDENCE FROM A MULTIETHNIC COHORT

**DOI:** 10.1093/geroni/igz038.3236

**Published:** 2019-11-08

**Authors:** Fadi Youkhana, Yanyan Wu, Catherine M Pirkle, Eric Hurwitz, Andrew Grandinetti, Alan Katz, Lynne R Wilkens

**Affiliations:** 1 University of Hawai’i at Mānoa, Honolulu, Hawai’i, United States; 2 University of Hawaii at Manoa, Hawaii, United States

## Abstract

Parkinson’s disease (PD) is the second most common neurodegenerative disease in the United States with more than 50,000 new cases annually. Studies have reported an inverse relationship between smoking status and the risk for PD. Current smoking status, the number of pack-years smoked, and the lifetime duration of smoking have all been shown to have a lower risk for PD compared to non-smokers. However, studies exploring smoking behaviors in a multiethnic cohort with an ample sample size of PD cases to analyze smoking differences between men and women are rare. Using the Multiethnic Cohort (MEC), our study included 680 self-reported cases of PD from total sample of 98,191 Blacks, Latinos, Japanese, Native Hawaiians, and Whites from Hawaii and Los Angeles surveyed in 2003-2007. Stratified by sex, we conducted a cross-sectional logistic regression analysis to examine the odds of developing PD by various smoking indicators. Overall, current smokers had the lowest risk for PD (OR=0.46, 95%CI 0.27-0.76) compared to non-smokers. The odds of developing PD gradually decreased as the number of years of smoking increased with participants that smoked for 50 years or more having the lowest odds of developing PD (OR= 0.41, 95%CI 0.22-0.78) compared to non-smokers. Using a multiethnic cohort, our analyses further supported the inverse association between PD and smoking status, as well as the number of years of smoking. Future studies are necessary to investigate the possible genetic modulation on the relationship between tobacco and PD.

